# Supplementary material for: Integrated solid-state NMR and molecular dynamics modeling determines membrane insertion of human β-defensin analog
Source: Commun Biol. 2019 Nov 1;2:402. doi: 10.1038/s42003-019-0653-6 (PMC6825183; doi:10.1038/s42003-019-0653-6)
Supplement: Supplementary file 1 — Supplementary Information [file 42003_2019_653_MOESM1_ESM.pdf]

Supplementary Information

Integrated Solid-State NMR and Molecular Dynamics Modeling Determines Membrane Insertion of Human β-Defensin Analog

Kang et al.

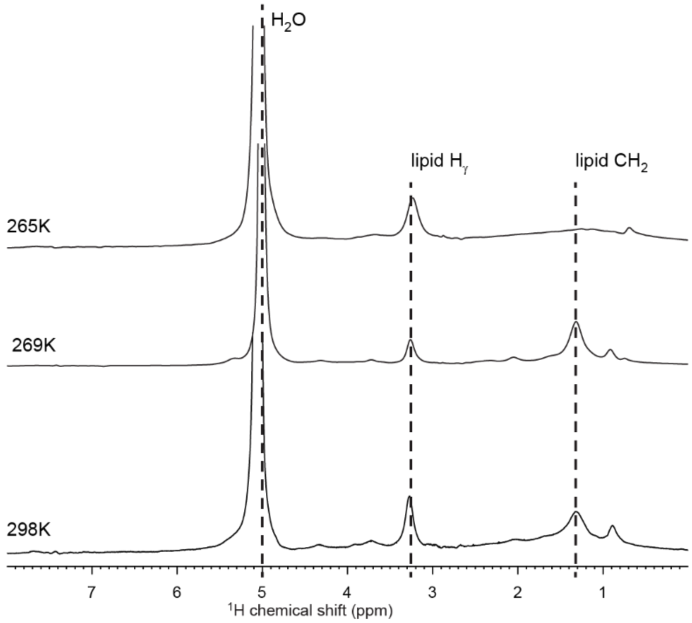

Supplementary Figure 1. 1D ^{1}H spectra of POPC/POPG membrane at various temperatures. The lipids are still in liquid crystalline phase at a thermocouple temperature of 269 K under 10 kHz MAS.

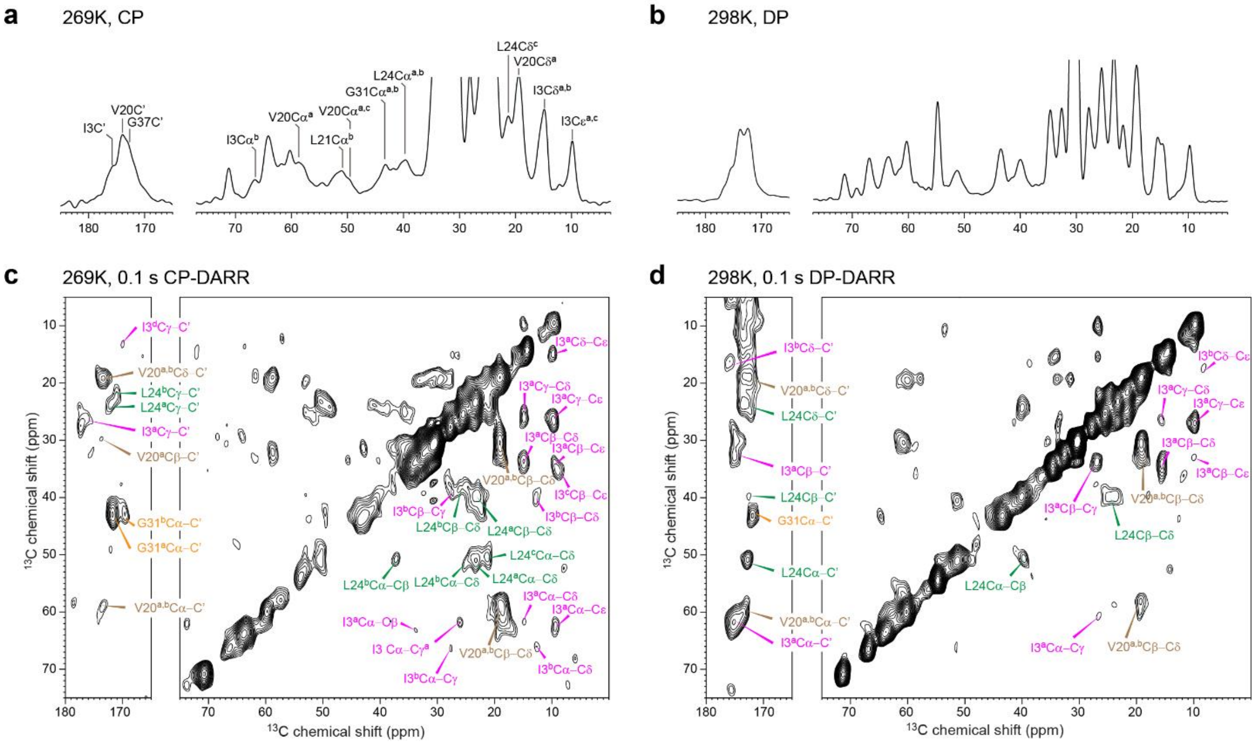

Supplementary Figure 2. ^{13}C spectra of IVLA peptide in POPC/POPG bilayers. a, 1D ^{13}C CP spectrum of IVLA at 269K. **b**, 1D ^{13}C DP spectrum of IVLA at 298K. **c**, 100 ms 2D ^{13}C-^{13}C CP-DARR spectrum of IVLA at 269K. **d**, 100 ms 2D ^{13}C-^{13}C DP-DARR spectrum of IVLA at 298K.

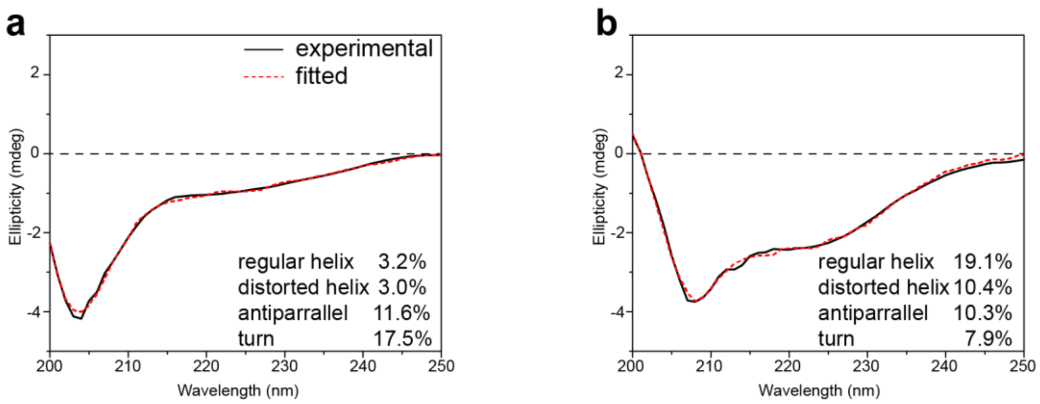

Supplementary Figure 3. CD spectra of hBD-3 analog with and without membranes. The spectra were measured in **a**, solution and **b**, POPC: POPG lipid bilayers at pH 7.0. Best-fit curves (red dash lines) are overlaid over the experimental spectra (black), and deconvolution results are presented. The spectrum of fully dissolved, solution state of hBD-3 analog shows minimal helix component, whereas the spectrum of membrane-bound state of hBD-3 reveals substantial amount of helix feature, suggesting large conformational change upon binding to membrane. The CD spectra reflect the average results of all the individual peptide state in each sample and does not provide conformer- or site-specific information.

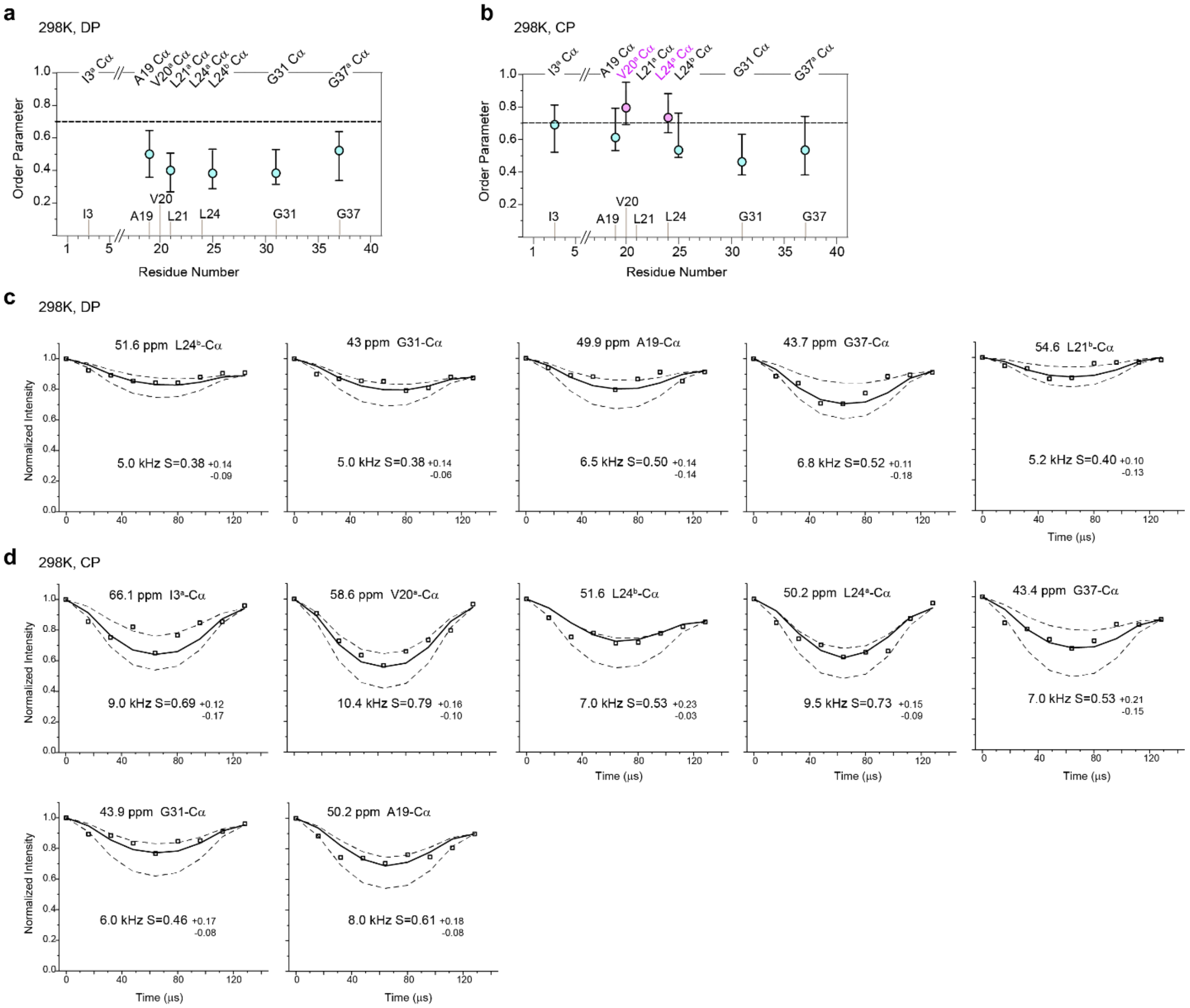

Supplementary Figure 4. Mobility of hBD-3 analog in POPC/POPG bilayers at 298K. a, Backbone Cα-Hα order parameters of the mobile-phase hBD-3 analog measured with DP at 298 K. **b**, Backbone Cα-Hα order parameters of membrane-bounded hBD-3 analog measured with CP at 298 K. The relatively rigid residues are highlighted in magenta and the mobile residues are in cyan. The $^{13}C-^{1}H$ dipolar couplings curves are shown in **c** and **d** for DP and CP measurements, respectively. The best-fit dipolar couplings, the corresponding order parameters, the error bars, and the T_2 relaxation times used to correct the asymmetry are labeled for each residue.

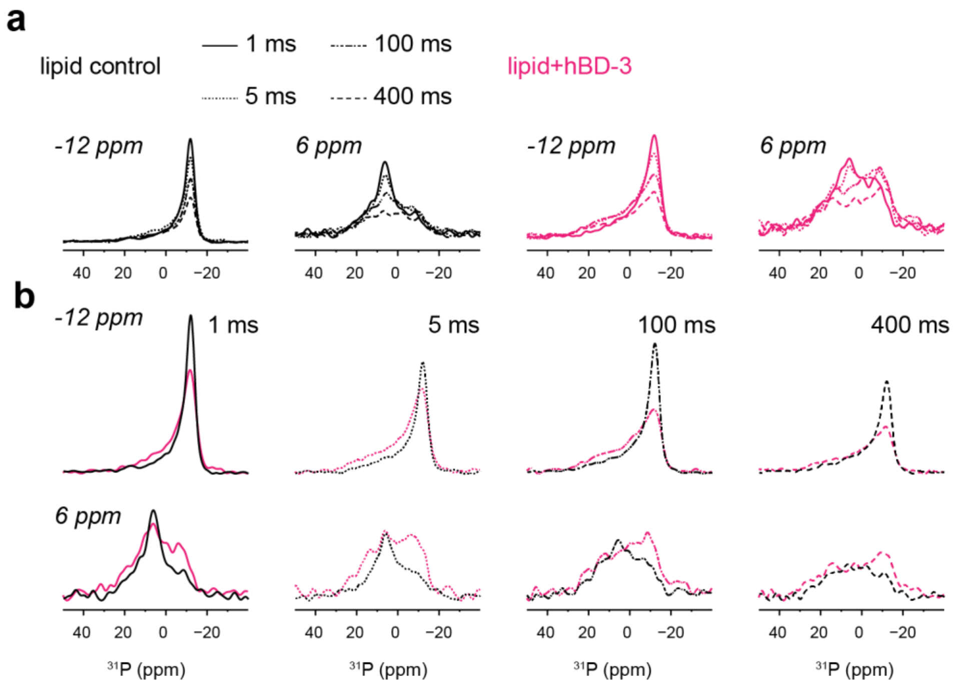

Supplementary Figure 5. Cross sections of 2D static ^{31}P-^{31}P exchange spectra. The POPC/G membranes without (black) and with (magenta) hBD-3 analog are compared. The cross sections are extracted from 6 ppm and -12 ppm to cover the range of the 2D spectra with mixing times of 1, 5, 100, and 400 ms. The intensity was scaled by **a,** number of scans and **b,** the samples amount ratio factor (0.75) obtained from the integral of 1D static ^{31}P spectrum.

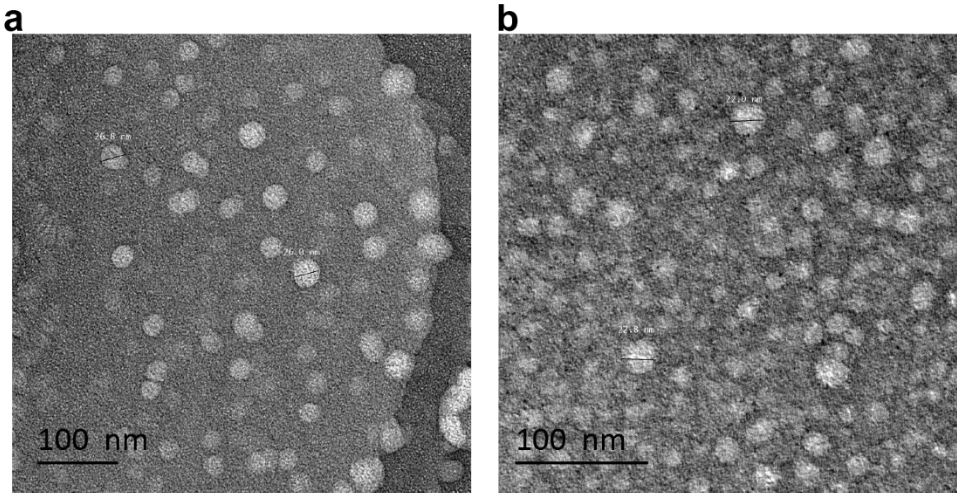

Supplementary Figure 6. Negatively stained TEM images of POPC/G membranes without (a) and with (b) hBD-3 analog. The peptide-containing vesicles are typically smaller and non-spherical.

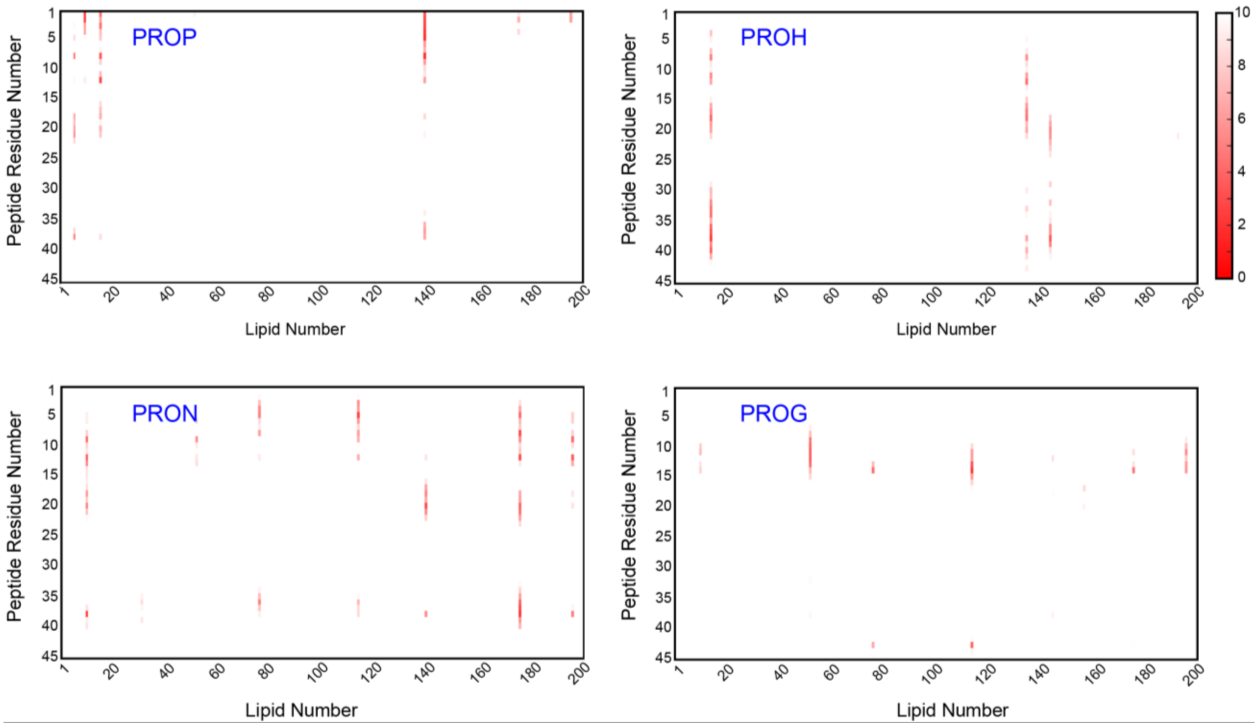

Supplementary Figure 7. Distance map between each single residue on peptide with lipid from 5.0 μs MD simulation. The minimum distance between each residue on peptide and each lipid was calculated, and then averaged based on the last 4.0us simulation trajectories. The average minimum distances from all the residue-lipid pairs comprises the distance map. The data is shown for peptide PROP, PROH, PRON and PROG. Binding distance being zero (red) indicates tight binding between a residue and the lipid. Any binding distances equal or larger than 10 Å will be shown in white. PROP, PROH and PRON form stable binding with the lipids consistently, while PROG residue 9-16 region binds with lipid membrane consistently. These data agree with the RMSF result shown in **Fig. 6e** and the insertion depth map result.

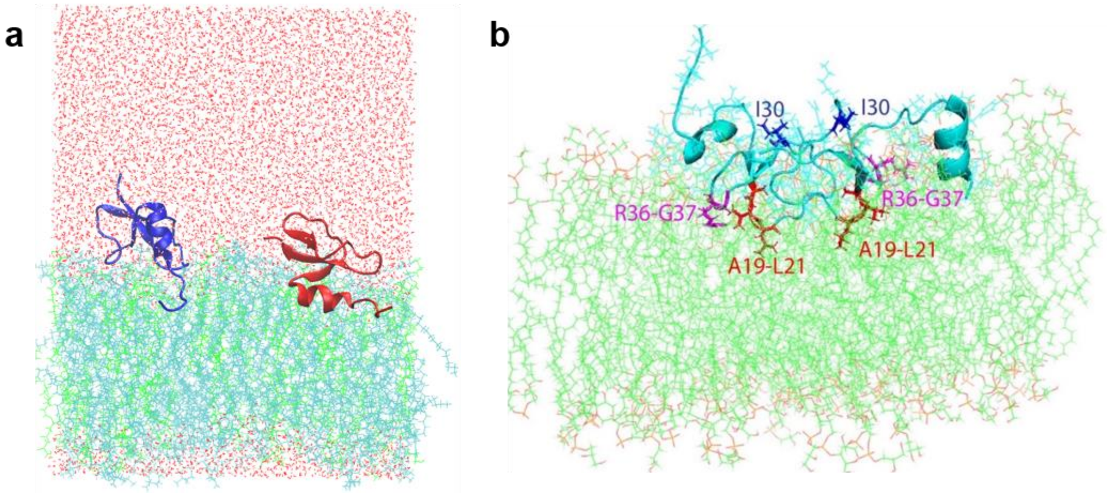

Supplementary Figure 8. The last structure of hBD-3 dimer in analog form in lipid bilayers. a, Dimer in POPC/G lipid bilayers. The structure is obtained with water and ions after 600 ns all atom NAMD self-assembly simulations. One unit of hBD-3 is shown in red cartoon, the other in blue cartoon. The POPC lipids are shown in cyan while POPG lipids in green. TIP3P water molecules are shown in red. **b,** hBD-3 analog dimer on pure POPG bilayers at 300 K for 300 ns. In total there are 144 POPG lipids including 72 POPG on the top layer and 72 POPG lipids on the bottom layer in the simulation. The NMR-detected such as I30, A19-L21, R36-G37 residues are shown in sticks. The I30 residues are shown in blue, A19-L21 in red and R36-G37 in magenta. The I30 residues are partially embedded on the dimer interface.

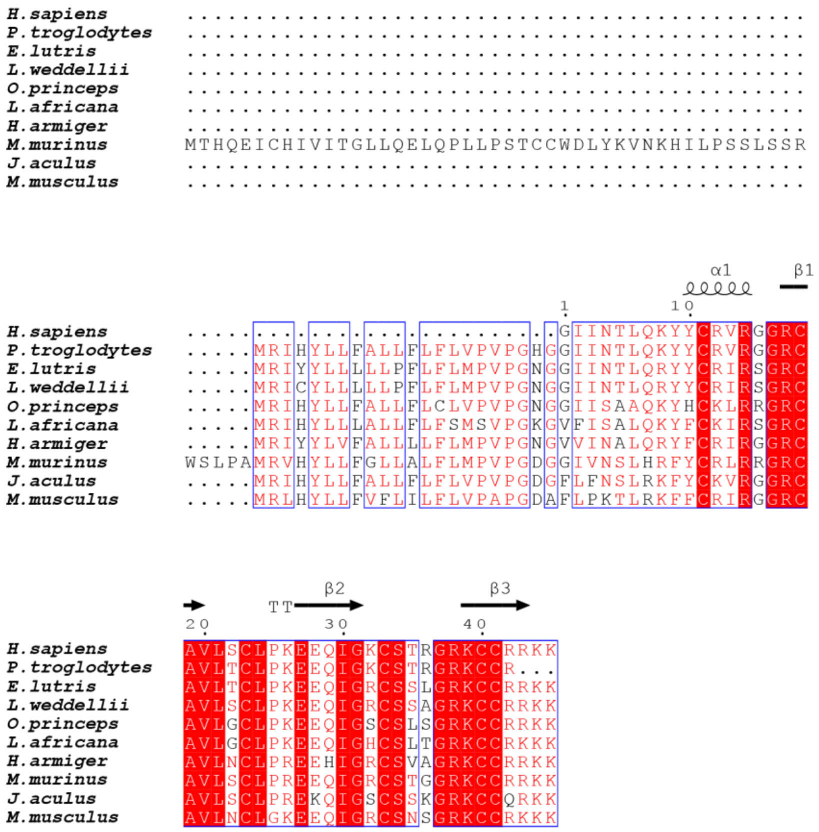

Supplementary Figure 9. Sequence alignment of hBD-3 with its homologues. The conserved residues are highlighted in red. The protein sequences were obtained from GenBank™. The alignment was obtained with the program Clustal W[1] and generated using ESPript server[2].

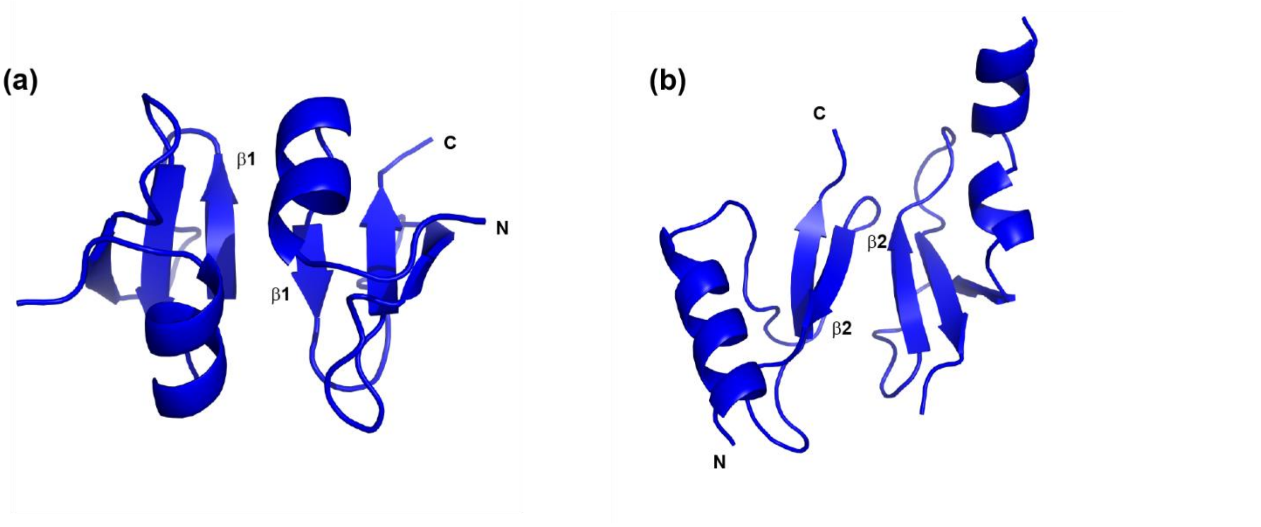

Supplementary Figure 10. Two proposed hBD-3 dimer models from literature. The models are proposed using **a**, x-ray and **b**, solution NMR evidence[3, 4, 5]. The original dimer structure used in the MD simulation of this study was a recent MD structure[6] that started from the structure in Supplementary **Fig. 10b**.

Supplementary Table 1. ^{13}C chemical shifts of dissolved and membrane-bound hBD-3. The major conformer is highlighted in bold. [1] from the VALIG sample, [2] from the IVLG sample. All ^{13}C chemical shifts are on the TMS scale.

Residue	CO	Cα	Cβ	Cγ	Cδ	Cε	Condition
[2]I3	**177.3**	**62.8**	**35.4**	**27.0**	**15.3**	**10.3**	269K, membrane-bound
	-	66.5	39.3	28.1	15.5	-	
	-	-	36.0	-	-	9.4	
[1]V13	**175.3**	**64.0**	**29.0**	-	**19.6**		
	173.8	59.2	33.0	-	19.4		
[1]A19	**174.6**	**50.2**	**17.3**				
	-	49.7	20.5				
[2]V20	173.8	58.9	30.6	-	19.3		
	174.4	61.5	32.4		18.8		
[1]L21	**174.7**	**51.6**	**-**		**23.8**		
	173.4	54.4	42.3		21.4		
	174.6	51.2	42.7		20.8		
[2]L24	172.6	50.6	40.9		23.6		
	-	51.7	38.1		25.1		
	-	50.4	43.5		21.7		
[1]I30	**174.9**	**59.9**	**37.0**	**25.2**	**15.6**	**-**	
	173.8	59.2	40.7	24.9	17.1	11.3	
	173.2	56.9	38.7	23.8	16.4	10.5	
[2]G31	**172.2**	**42.8**					
	170.1	42.8					
[1]G37	**171.8**	**43.8**					
	171.5	46.3					
[2]I3	**176.3**	**61.2**	**33.3**	**27.4**	**15.8**	**10.0**	298K, in solution
	175.8	-	-	-	18.1	8.4	
[1]V13	174.5	62.7	29.9	-	20.2		
[1]A19	**175.5**	**50.6**	**17.6**				
	-	50.6	20.1				
[2]V20	**174.0**	**60.5**	**31.6**	**-**	**19.9**		
	173.5	58.0	34.0	-	19.2		
[1]L21	175.3	53.4	41.7		24.6		
[2]L24	173.1	51.0	40.1		24.4		
[1]I30	**175.5**	**59.5**	**37.1**	**25.4**	**15.9**	**11.2**	
	173.9	-	40.3	25.3	-	-	
[2]G31	172.3	43.1					
[1]G37	172.3	43.7					

Supplementary Table 2. Summary of dipolar order parameters of hBD-3 analogs. The experiments are detected using CP and DP at different temperatures. The carbon site, chemical shift, best-fit CH dipolar coupling, order parameter, error bars, and T_2 relaxation times used to correct the asymmetry are listed.

Experiment condition	Carbon site	Chemical shift (ppm)	Best-fit CH/CH_2 dipolar coupling (kHz)	Order parameter	Error bars	T_2 relaxation times used (ms)
269K, CP	I3-Cα	66.2	6.8	0.52	+ 0.05<br>- 0.09	5.0
	A19-Cα	50.2	8.4	0.64	+ 0.01<br>- 0.03	1.8
	V20-Cα	58.2	10.6	0.82	+ 0.09<br>- 0.01	1.1
	L21-Cα	54.0	6.5	0.50	+ 0.11<br>- 0.02	1.1
	L24[a]-Cα	49.8	10.0	0.76	+ 0.08<br>- 0.10	1.0
	L24[b]-Cα	51.6	9.5	9.73	+ 0.01<br>- 0.12	1.2
	G31-Cα	42.5	8.7	0.66	+ 0.07<br>- 0.03	1.1
	G37-Cα	43.7	10.0	0.73	+ 0.05<br>- 0.03	1.1
298K, CP	I3[a]-Cα	66.1	9.0	0.69	+ 0.12<br>- 0.17	2.1
	A19-Cα	50.2	8.0	0.61	+ 0.18<br>- 0.08	1.2
	V20[a]-Cα	58.6	10.4	0.79	+ 0.16<br>- 0.10	2.3
	L24[a]-Cα	50.2	9.5	0.73	+ 0.15<br>- 0.09	2.1
	L24[b]-Cα	51.6	7.0	0.53	+ 0.23<br>- 0.03	0.8
	G31-Cα	43.9	6.0	0.46	+ 0.17<br>- 0.06	2.5
298K, DP	A19-Cα	49.9	6.5	0.5	+ 0.14<br>- 0.14	1.5
	L21[b]-Cα	54.6	5.2	0.4	+ 0.10<br>- 0.13	0.0
	L24[b]-Cα	51.6	5.0	0.38	+ 0.14<br>- 0.09	1.1
	G31-Cα	43.0	5.0	0.38	+ 0.14<br>- 0.06	1.0
	G37-Cα	43.9	6.8	0.52	+ 0.11<br>- 0.18	1.5

Supplementary Table 3. Chemical Shift Anisotropy (CSA) parameters for POPC and POPG lipids. The data are obtained using the sideband intensities of 1D ^{31}P spectra collected under 3.5 kHz slow MAS. Both Peptide-free and peptide-containing samples are measured.

			δ_{11}	δ_{22}	δ_{33}	Span	Asymmetry
^{31}P-CP	POPC	+hBD3	22.53	-12.43	-12.56	35.09	0.01
		control	22.71	-9.73	-15.44	38.15	0.24
	POPG	+hBD3	23.22	-11.17	-11.34	34.56	0.01
		control	19.25	-9.26	-9.26	28.51	0.00
^{31}P-DP	POPC	+hBD3	20.94	-9.05	-14.35	35.29	0.01
		control	25.48	-13.86	-14.09	39.57	0.24
	POPG	+hBD3	23.77	-11.45	-11.59	35.36	0.01
		control	19.87	-9.57	-9.57	29.44	0.00

Supplementary Table 4. Solvent accessible surface area (SASA) of hBD-3 labeled residues. The data are obtained from the monomeric solution structure. The SASA range from 2 to 140 Angstroms^2 for all the labeled residues. A19 and G31 having the lowest SASA values; the small SASA values indicate the sidechains of those three residues point to the protein hydrophobic core and stay buried in the monomeric structure. I30, G37 and L24 has moderate SASA while I3, V20 and L21 has large SASA values.

Residue	Solvent Accessible Surface Area (Angstroms^2)
I3	140.179
A19	6.234
V20	113.303
L21	136.369
L24	75.139
I30	30.402
G31	1.625
G37	48.106

Supplementary References

1. Thompson JD, Higgins DG, Gibson TJ. Clustal-W - Improving the Sensitivity of Progressive Multiple Sequence Alignment through Sequence Weighting, Position-Specific Gap Penalties and Weight Matrix Choice. *Nucleic Acids Res* **22**, 4673-4680 (1994).
2. Gouet P, Courcelle E, Stuart DI, Metoz F. ESPript: analysis of multiple sequence alignments in PostScript. *Bioinformatics* **15**, 305-308 (1999).
3. Hoover DM, Chertov O, Lubkowski J. The Structure of human β-defensin-1 new insights into structural properties of β-defensins. *J Biol Chem* **276**, 39021-39026 (2001).
4. Schibli DJ, *et al.* The solution structures of the human β-defensins lead to a better understanding of the potent bactericidal activity of HBD3 against Staphylococcus aureus. *J Biol Chem* **277**, 8279-8289 (2002).
5. Zimmermann GR, Legault P, Selsted ME, Pardi A. Solution Structure of Bovine Neutrophil Beta-Defensin-12 - the Peptide Fold of the Beta-Defensins Is Identical to That of the Classical Defensins. *Biochemistry-Us* **34**, 13663-13671 (1995).
6. Zhang L. Different dynamics and pathway of disulfide bonds reduction of two human defensins, a molecular dynamics simulation study. *Proteins* **85**, 665-681 (2017).
