# Supplementary material for: Integrated solid-state NMR and molecular dynamics modeling determines membrane insertion of human β-defensin analog
Source: Commun Biol. 2019 Nov 1;2:402. doi: 10.1038/s42003-019-0653-6 (PMC6825183; doi:10.1038/s42003-019-0653-6)
Supplement: Supplementary file 2 — Description of Additional Supplementary Files [file 42003_2019_653_MOESM2_ESM.docx]

**Supplementary Data 1**

**Solid-State NMR and MD Modeling Source File:** This Excel file contains source data associated with the solid-state NMR structural analysis shown in Figures 2a, 2b, 3b–d, 4b, 4c, and Supplementary Figures 4c and 4d. The residues, carbon sites, NMR time constants, and standard deviation error bars propagated from NMR signal-to-noise ratios are listed in the excel file. The Excel file also contains source data associated with the MD modeling results shown in Figures 6d, 6e, and Supplementary Figure 7. The RMSF, insertion depth matrix, and distance map data for each single residue (45 residues) of each peptide are listed.
